# The Expression of Tripartite Motif Protein 36 and *β*-Catenin Correlates with the Prognosis of Esophageal Cancer

**DOI:** 10.1155/2020/7641761

**Published:** 2020-07-31

**Authors:** Hua Zhang, Wenlong Sun, Gaofeng Qiao, Bin Zhao, Xiangyan Liu, Feng Zhu

**Affiliations:** ^1^Department of Thoracic Surgery, Shandong Provincial Chest Hospital, Cheeloo College of Medicine, Shandong University, Jinan, Shandong 250013, China; ^2^Department of Thoracic Surgery, Boxing People's Hospital of Shandong Province, Binzhou, Shandong 256500, China; ^3^Department of Thoracic Surgery, Shandong Provincial Hospital, Cheeloo College of Medicine, Shandong University, Jinan, Shandong 250021, China

## Abstract

**Aims:**

Tripartite motif protein 36 (TRIM36) plays a tumor-suppressive role in prostate cancer. However, there is little information on the clinical relevance of TRIM36 expression in esophageal cancer (ESCA).

**Methods:**

TRIM36 expression was analyzed by using The Cancer Genome Atlas (TCGA) ESCA dataset as well as by quantitative real-time polymerase chain reaction (PCR) and immunohistochemical (IHC) staining on samples from our hospital.

**Results:**

In the current study, the analysis of TCGA ESCA dataset suggested the decreased expression of TRIM36 in ESCA tissues. Further analyses on samples from our hospital demonstrated that TRIM36 was significantly downregulated in ESCA tissues than in the noncancerous controls at both the mRNA and protein levels. Moreover, gene set enrichment analysis on TCGA ESCA dataset suggested that TRIM36 expression was inversely correlated with the *β*-catenin pathway. IHC staining data showed that 66.25% (53/80) and 51.25% (41/80) of ESCA cases had a low expression of TRIM36 and a high expression of *β*-catenin, respectively. By Fisher's exact test, we found that TRIM36 protein expression was significantly correlated with tumor size (*P* = 0.0104), tumor stage (*P* = 0.0169), lymph node metastasis (*P* = 0.0021), vital status (*P* = 0.0443), and *β*-catenin expression (*P* = 0.0329). These findings suggest the potential clinical significance of TRIM36 in ESCA. Kaplan–Meier and log-rank test demonstrated that a low expression of TRIM6 and a high expression of *β*-catenin were associated with poor overall survival of ESCA patients.

**Conclusions:**

Our study provides evidence for the prognostic value of TRIM36 in ESCA.

## 1. Introduction

Esophageal cancer (ESCA) is one of the most common malignancies worldwide [1]. Esophageal squamous-cell carcinoma (ESCC) and esophageal adenocarcinoma (EAC) are the two main subtypes of this disease [2]. Despite recent advances in multimodal treatment combining esophagectomy with chemotherapy, radiation, and targeted therapies, the prognosis of ESCA is still not satisfactory due to the aggressiveness of this disease [3, 4]. Therefore, novel biomarkers for ESCA need to be urgently identified to aid in the improvement of therapeutic strategies and the clinical outcome.

The Wnt signaling pathway, a homolog of the Wingless (Wg) signaling pathway in Drosophila, regulates embryonic development, cell proliferation, differentiation, etc. via a wide spectrum of target genes [5]. *β*-Catenin is the key control protein in the canonical Wnt pathway. The Wnt signaling pathway transmits signals by inducing the accumulation of *β*-catenin in the cytoplasm and nuclei [6]. When the Wnt/*β*-catenin pathway is deregulated, abnormal cell growth and cancer are promoted [5]. The abnormally constitutive activation of the Wnt/*β*-catenin pathway is frequently detected in various human cancers, including ESCA [7, 8].

Tripartite motif (TRIM) proteins are characterized by a common domain structure consisting of a RING finger, one or two zinc-binding motifs known as B-boxes, and a coiled-coil motif [9]. TRIM family proteins have been implicated in a wide range of biological processes, and alterations of TRIM proteins often result in a variety of pathological conditions such as viral infection, genetic diseases, and carcinogenesis [9–11]. TRIM36, as a member of the TRIM family, is mapped to chromosome 5q22.3 [12]. It has been reported that TRIM36 is a microtubule-binding protein and plays a role in embryogenesis, acrosome reaction, chromosome segregation, and cell cycle progression [13–15]. Recently, TRIM36 has been identified as an androgen-responsive gene, and its tumor-suppressive role has been reported in prostate cancer [16, 17]. Other members of the TRIM protein family have been reported to play an important role in human cancers through the Wnt/*β*-catenin pathway. The interactions of the Wnt/*β*-catenin pathway and other members of the TRIM protein family, such as TRIM29 [18, 19], TRIM28 [20], TRIM32 [21], TRIM33 [22], and TRIM44 [23], may play important roles in the development of human cancers. However, the correlation between TRIM36 and *β*-catenin has not yet been studied.

In the present study, we analyzed a public ESCA dataset and found a significant downregulation of TRIM36 in ESCA samples and a negative correlation between TRIM36 expression and the *β*-catenin pathway. Subsequently, immunohistochemical staining was performed to assess the expression of TRIM36 and *β*-catenin in ESCA. Finally, we investigated the possible prognostic value of TRIM36 and *β*-catenin expression in ESCA by analyzing the relationship between TRIM36 protein expression and the clinicopathological parameters.

## 2. Materials and Methods

### 2.1. Bioinformatics Analysis

The gene expression data were obtained from The Cancer Genome Atlas website (TCGA, https://tcga-data.nci.nih.gov/tcga/) for ESCA. Gene set enrichment analysis (GSEA) was performed based on TCGA ESCA dataset using the GSEA version 2.0 according to Subramanian et al. [24].

### 2.2. Tissue Specimens

The Institutional Review Board of the Shandong Provincial Hospital Affiliated to the Shandong University approved this study. Eighty patients who underwent surgical resection at the Shandong Provincial Hospital Affiliated to the Shandong University (China) between February 2009 and May 2010 (48 men and 32 women) were enrolled in this study after written informed consents were obtained. No patients received any preoperative chemotherapy or radiotherapy. ESCA and normal esophageal mucosa tissues collected from all the patients were formalin fixed, paraffin embedded, cut into 4 m thick sections, and used for immunohistochemical (IHC) staining of TRIM36 and *β*-catenin. Of these samples, 27 pairs of ESCA and normal esophageal mucosa samples were stored at −80°C and were available for quantitative real-time PCR analysis.

### 2.3. RNA Isolation and Quantitative Real-Time PCR (QRT-PCR)

Total RNA was isolated from the tissue samples with TRIzol (Invitrogen Carlsbad, CA, USA) following the manufacturer's protocol. qRT-PCR was performed to examine the expression levels of TRIM36 mRNA with the SYBR® Green kit (Thermo Fisher Scientific) on ABI 7300 instrument (Applied Biosystems). GAPDH was amplified as an internal control. Dissociation curve analysis was performed at the end of PCR cycles to verify specific product amplification. The relative expression of TRIM36 was calculated by the formula 2^‐△△^CT. The primers are as follows: TRIM36, 5′-CGTCGGTCCTCCAGAGTTTGTG-3′ and 5′-GTGGCAAGTTCCGTCGTCTTCC-3′; GAPDH, 5′-AATCCCATCACCATCTTC-3′ and 5′-AGGCTGTTGTCATACTTC-3′.

### 2.4. IHC Analysis

A standard IHC technique was performed to determine the expression of TRIM36 and *β*-catenin in tissue samples. After deparaffinization and rehydration, antigen retrieval was performed with 0.01 M citrate buffer (pH 6.0) in a microwave for 15 min. Following the inactivation of endogenous peroxidases and the blockage of nonspecific antigens, the sections were incubated with anti-TRIM36 (Abcam, Cambridge, MA, USA) or anti-*β*-catenin (Bioss Inc., Woburn, Massachusetts, USA) overnight at 4°C. After washing, the slides were probed with horseradish peroxidase- (HRP-) conjugated secondary antibody for 1 h at room temperature, treated with the 3,3-diaminobenzidine (DAB) solution, and counterstained with hematoxylin. The slides were reviewed independently by two pathologists. The immunoreactive score (IS) was assessed by using the following formula: IS = percentage (0, no positive cells; 1, 1%–10%; 2, 11%–50%positive; 3, >50%positive) × staining intensity (0, absent; 1, weak; 2, moderate; 3, strong). The target protein was classified as high expression when IS ranged from 3 to 9, otherwise classified as low expression.

### 2.5. Statistical Analysis

Statistical analysis was performed using the GraphPad Prism software (San Diego, CA, USA). Student's *t*-test was performed to examine the differences between groups. Fisher's exact test was used to determine the correlation between TRIM36 protein expression and clinicopathological features, such as gender, age, tumor size, TNM stage, lymph node metastasis, and vital status. Kaplan–Meier survival plots and log-rank analysis were carried out to determine the difference in survival. A *P* value <0.05 was deemed significant.

## 3. Results

### 3.1. TRIM36 Expression Was Downregulated in ESCA

**Figure 1:**
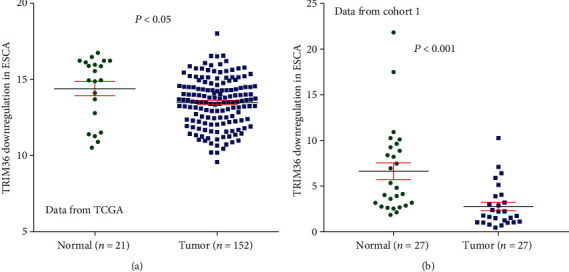
TRIM36 mRNA expression was downregulated in ESCA tissues. (a) The expression level of TRIM36 in ESCA and normal tissues based on TCGA ESCA dataset. (b) The mRNA levels of TRIM36 in 27 paired ESCA and nontumorous tissues were determined using qRT-PCR.

By analyzing data from TCGA ESCA, we found that TRIM36 mRNA expression was significantly decreased in ESCA samples as compared with normal esophageal mucosa specimens (*P* < 0.05, Figure 1(a)). Furthermore, qRT-PCR analysis was performed on 27 pairs of primary ESCA and normal esophageal mucosal samples from our hospital. Evidently, TRIM36 expression was downregulated at the RNA level in the tested specimens (*P* < 0.001, Figure 1(b)).

### 3.2. Decreased Expression of TRIM36 Correlated with Clinical Parameters of ESCA

**Figure 2:**
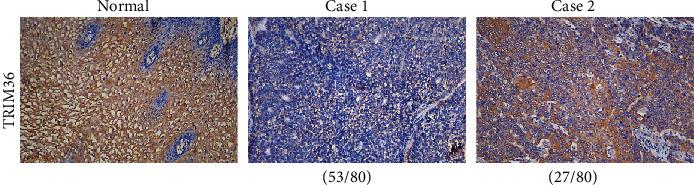
Protein expression of TRIM36 was downregulated in ESCA tissues. Expression of TRIM36 was determined by IHC staining in ESCA tissues (53 low expression and 27 high expression). Magnification: 200x.

The protein levels of TRIM36 were detected in 80 cases of ESCC and normal tissues by IHC staining. The clinical characteristics of these patients are listed in Table 1. TRIM36 expression was observed in the cytoplasm and was lower in ESCA compared with normal tissues (Figure 2). Of the 80 patients, 66.25% (53 cases) and 33.75% (27 cases) showed low and high expression of TRIM36, respectively.

**Figure 3:**
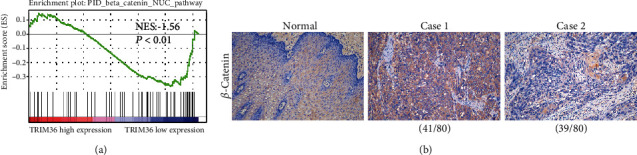
Protein expression of *β*-catenin in ESCA tissues. (a) GSEA was performed using TCGA ESCA dataset. The *β*-catenin pathway was strongly associated with TRIM36 expression. (b) Immunohistochemical staining of *β*-catenin in ESCA tissues (41 high expression and 39 low expression). Magnification: 200x.

Furthermore, GSEA analysis showed that TRIM36 expression was negatively correlated with the *β*-catenin pathway (Figure 3(a)). Thus, the protein expression of *β*-catenin was then determined in the same 80 cases by IHC. We found that *β*-catenin was expressed in the cytoplasm and nuclei (Figure 3(b)) and high and low expressions of *β*-catenin were observed in 48.75% (39 cases) and 51.25% (41 cases) of the tested ESCA specimens, respectively.

In addition, we analyzed the correlation between TRIM36 expression and the clinical parameters of ESCA using Fisher's exact test. As shown in Table 2, TRIM36 protein expression was correlated with tumor size (*P* = 0.0104), tumor stage (*P* = 0.0169), lymph node metastasis (*P* = 0.0021), vital status (*P* = 0.0443), and *β*-catenin expression (*P* = 0.0329). These findings suggest the potential clinical significance of TRIM36 in ESCA.

### 3.3. Decreased Expression of TRIM36 Was Closely Related to the Poor Prognosis of Patients with ESCA

**Figure 4:**
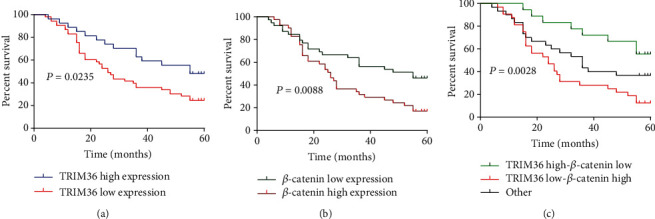
Decreased expression of TRIM36 was closely related with the poor prognosis of patients with ESCA. Kaplan–Meier survival plots in ESCA. The patients with ESCA (*n* = 80) were divided into different groups based on (a) TRIM36 expression, (b) *β*-catenin expression, (c) TRIM36, and *β*-catenin coexpression evaluated by immunohistochemical staining.

The correlation between the expression of both proteins and the overall survival of patients with ESCA was also explored. Kaplan–Meier survival curves showed that patients with low TRIM36 expressions (*P* = 0.0235; Figure 4(a)) and high *β*-catenin expressions (*P* = 0.0088; Figure 4(b)) had a marked decreased survival time as compared to those with high expressions of TRIM36 and low expressions of *β*-catenin, respectively. Furthermore, patients with low expressions of TRIM36 and high expressions of *β*-catenin had the shortest overall survival, whereas those with high expressions of TRIM36 and low expressions of *β*-catenin had the longest overall survival time (*P* = 0.0028; Figure 4(c)).

## 4. Discussion

It has been reported that negative TRIM36 expression could be used as a prognostic indicator of the biochemical recurrence-free survival in prostate cancer [16]. In the current study, we initially reported that both mRNA and protein expression of TRIM36 were decreased in ESCA tissues. The protein expression of TRIM36 was closely related to tumor size, tumor stage, lymph node metastasis, and vital status. Compared with patients with high expressions of TRIM36, the survival time of patients with low expressions of TRIM36 was significantly shortened. These data suggest that TRIM36 expression may be used as a novel prognostic indicator for ESCA, although further studies with a larger sample size are needed to validate these findings. A previous study has linked TRIM36 expression to cell cycle progression and proliferation in NIH3T3 cells [15] and prostate cancer cells [16]. We speculated that TRIM36 may play a role in the development of ESCA through regulating cell growth, which will be explored in the future.

Currently, studies have reported that the abnormal expression of *β*-catenin is associated with esophageal tumorigenesis [7, 8]. In corroboration with the previous findings, the present study showed that the survival time of patients with ESCA was correlated with *β*-catenin expression, suggesting the prognostic values of *β*-catenin in ESCA. Several studies have suggested the interactions of other members of TRIM proteins and the Wnt/*β*-catenin pathway in human cancers. For example, Zhou et al. found that TRIM29 positively correlates with the abnormal expression of *β*-catenin in lung squamous-cell carcinoma, but not in lung adenocarcinoma [18]. Wang et al. showed the correlation between TRIM29 and *β*-catenin levels in pancreatic cancer, and their study also indicated that the oncogenic effects of TRIM29 is dependent on *β*-catenin function [19]. Xue et al. reported that TRIM33 levels are inversely correlated with *β*-catenin in glioblastoma samples and that TRIM33 plays a tumor-suppressive role by degrading nuclear *β*-catenin [22]. TRIM32 [21], TRIM28 [20], and TRIM44 [23] promote cancer progression by activating *β*-catenin signaling. In this study, GSEA analysis suggested a negative correlation between TRIM36 expression and the *β*-catenin pathway in TCGA ESCA dataset. We then provided evidence for the negative correlation in protein levels by immunohistochemical staining. Furthermore, patients with high expressions of TRIM36 and low expressions of *β*-catenin showed the best overall survival, whereas patients with low expressions of TRIM36 and high expressions of *β*-catenin displayed the worst overall survival. Therefore, the current study suggests that TRIM36 and *β*-catenin may cooperate in ESCA. TRIM36 has ubiquitin E3 ligase activity [15]. The mechanisms how TRIM36 cooperated with *β*-catenin are to be investigated in the future.

In summary, we have shown for the first time the correlation between TRIM36 and *β*-catenin expression in ESCA. TRIM36 expression may be used as a novel prognostic indicator for ESCA. The biological functions of TRIM36 in the development of ESCA still warrant further investigation.

## Figures and Tables

**Table 1:** Clinicopathological characteristics in ESCA patients (*n* = 80).

Parameters		Cases	%
Age (years)	<60	41	51.25
	≥60	39	48.75
Gender	Male	48	60
	Female	32	40
Tumor size	<4 cm	43	53.75
	≥5 cm	37	46.25
TNM stage	I + II	47	58.75
	III	33	41.25
Lymphnode metastasis	Absent	46	57.5
	Present	34	42.5
Vital status	Alive	26	32.5
	Dead	54	67.5
TRIM36 expression	Low	53	66.25
	High	27	33.75
*β*-Catenin expression	Low	39	48.75
	High	41	51.25

**Table 2:** Correlation between TRIM36 expression and clinicopathologic factors of ESCA patients.

Parameters		All cases	TRIM36 expression		*P* value
			Low (*n* = 53)	High (*n* = 27)	
Age (years)	<60	41	26	15	0.6410
	≥60	39	27	12	
Gender	Male	48	34	14	0.3388
	Female	32	19	13	
Tumor size	<4 cm	43	23	20	0.0104^∗^
	≥5 cm	37	30	7	
TNM stage	I + II	47	26	21	0.0169^∗^
	III	33	27	6	
Lymphnode metastasis	Absent	46	24	22	0.0021^∗∗^
	Present	34	29	5	
Vital status	Alive	26	13	13	0.0443^∗^
	Dead	54	40	14	
*β*-Catenin expression	Low	39	21	18	0.0329^∗^
	High	41	32	9	

Fisher's exact test, ^∗^*P* < 0.05 and ^∗∗^*P* < 0.01.

## Data Availability

The data used to support the findings of this study are available from the corresponding author upon request.
